# MECT-MobileViT: A Lightweight Fish Weight Prediction Model Based on Dual-View Morphological Feature Fusion and Anti-Interference Attention

**DOI:** 10.3390/ani16132076

**Published:** 2026-07-05

**Authors:** Yi Wang, Mingyu Tan, Jingtao Deng, Lin Yang, Yongjie Wu, Hao Peng, Cheng Ouyang, Yahui Luo, Wenwu Hu, Pin Jiang

**Affiliations:** 1 College of Information and Intelligence, Hunan Agricultural University, Changsha 410128, China; wangyi@hunau.edu.cn (Y.W.); 19907426171@stu.hunau.edu.cn (M.T.); 984711035@stu.hunau.edu.cn (J.D.); 2767959117@stu.hunau.edu.cn (L.Y.); wyj@stu.hunau.edu.cn (Y.W.); sx20230201@stu.hunau.edu.cn (H.P.); ouyang@stu.hunau.edu.cn (C.O.); 2 College of Mechanical and Electrical Engineering, Hunan Agricultural University, Changsha 410128, China; luoyh@hunau.edu.cn

**Keywords:** body weight prediction, model lightweighting, deep learning, multi-scale fusion, dual-view

## Abstract

In intensive aquaculture, non-invasive real-time monitoring of morphological traits and body weight of largemouth bass (*Micropterus salmoides*) is essential for precision feeding and yield estimation. Manual measurement is laborious and stressful, whereas vision-based methods are challenged by insufficient dual-view feature fusion, poor robustness to underwater noise, and over-parameterized models unsuitable for edge deployment. To address these issues, a lightweight framework, MECT-MobileViT, is proposed based on MobileViT-xxs. A Morphometric-Guided Multi-Scale Fusion module is designed to couple physical priors with dual-branch visual features, strengthening shape–weight association. An ECA-NL attention block employing instance normalization, GLU gating, and threshold filtering is embedded to enhance feature robustness against visual disturbances typical in aquaculture and to accentuate critical morphological features. A three-stage synergistic pruning strategy—attention head pruning, structured channel pruning, and depthwise separable attention substitution—is applied to achieve substantial compression while preserving representational capacity. Experiments on a self-built lateral–dorsal dual-view dataset show that the proposed model significantly outperforms mainstream benchmarks. The pruned version attains an R^2^ of 0.8266 and an RMSE of 16.4201, with less than 2% accuracy degradation relative to the best unpruned model, and contains only 7.34 M parameters. This study demonstrates a promising prototype for contactless, stress-free weight estimation in largemouth bass and offers new technical insights into feature fusion, noise suppression, and collaborative model compression for aquaculture visual perception.

## Simple Summary

In aquaculture, monitoring the body weight of largemouth bass typically relies on manual capture and contact-based weighing, a practice that is inefficient and induces stress and injury. The objective of this study was to develop a non-destructive, rapid, and accurate automatic weight estimation method. A lightweight intelligent model was designed using dual-view images (lateral and top views) of the fish. Morphological information—including body length, body height, and body width—was fused, and resistance to underwater disturbances such as turbidity and debris was enhanced, thereby achieving accurate weight prediction. The model maintained high prediction accuracy while its size was substantially compressed, demonstrating the feasibility of a lightweight approach for potential edge deployment in farming environments. If validated on larger and more diverse datasets, this prototype could provide a convenient weight monitoring tool for farmers, facilitate precision feeding, reduce feed waste and fish stress, and promote the intelligent and sustainable development of aquaculture.

## 1. Introduction

Global aquaculture is transitioning rapidly toward intensive and intelligent production systems [1,2], and the non-destructive, real-time, and accurate prediction of live fish body weight has become a core technology underpinning the efficient operation of smart fisheries [3]. Recent advances in intelligent aquaculture monitoring, including cross-modal approaches that integrate visual and acoustic signals for behavioral assessment [4], further highlight the industry’s shift toward automated, non-invasive sensing solutions. Largemouth bass (*Micropterus salmoides*), a major freshwater aquaculture species in China, reached a production volume exceeding 750,000 tons in 2024, of which Guangdong Province accounted for 50.9%, with yields surpassing 4000 kg per mu reported in certain intelligent farming bases [5]. Nevertheless, body weight monitoring still relies on manual capture and contact-based measurement, which is characterized by low efficiency and strong stress induction, thereby constituting a bottleneck that restricts precision feeding, cost reduction, and efficiency improvement [6]. Consequently, vision-based, non-contact body weight monitoring is of considerable engineering significance for optimizing feed utilization, supporting fine-scale management, and promoting the sustainable development of the industry [7,8].

For largemouth bass, the limitations of manual measurement are particularly pronounced due to the species’ biological characteristics. The fish are aggressive and exhibit intense stress responses; manual capture and handling readily provoke jumping and struggling, which can cause physical injury and secondary infection [9,10,11]. It has been demonstrated that such stress responses can reduce growth rates by 10–15% and may even trigger mass mortality [12]. Moreover, largemouth bass are typically cultured at high densities. Using a representative 50-mu intensive pond as an example, manual sampling and measurement can require three to five workers and more than four hours to complete, and data accuracy is compromised by operator experience, meaning that the real-time monitoring demanded by precision feeding and refined management cannot be satisfied [13,14].

Early investigations into weight prediction for largemouth bass mostly constructed traditional mathematical regression models based on manually measured body length, body width, and body height. Xu et al. [15] adopted multiple linear regression, but Feng et al. [16] pointed out that such models can only fit simple linear relationships and cannot characterize the complex nonlinear associations between body weight and the species’ laterally compressed body shape, nor account for individual differences in fatness and condition. Additionally, these models still depend on manually acquired morphological data, and Wang et al. [17] reported that their errors generally exceeded 8%, indicating an inability to accommodate weight prediction requirements across different growth stages.

With the advancement of deep learning, vision-based weight estimation has progressed through three stages: traditional image processing, convolutional neural network (CNN) feature learning, and Vision Transformer global modeling [18]. Among traditional approaches, Er et al. [19] relied on hand-crafted features such as contours and area, but robustness was severely compromised under turbidity, uneven illumination, and variable postures. Yin et al. [20] and Hou et al. [21] employed CNNs to achieve end-to-end feature extraction, markedly improving detection accuracy. However, deep CNN models possess a vast number of parameters, making them difficult to adapt to farm-level edge devices. The similarity-aware Transformer proposed by Li et al. [22] exhibits excellent global feature modeling capability, yet its high computational complexity and slow inference speed prevent it from meeting the real-time monitoring demands of high-density culture environments.

To reconcile accuracy with efficiency, lightweight architectures and model compression strategies have been widely explored. MobileNet [23], ShuffleNet [24], and MobileViTv3 [25] demonstrate that lightweight CNN–Transformer hybrids are viable for aquatic vision tasks. In model compression, structured pruning techniques have achieved substantial parameter reduction with minimal accuracy loss [26,27]. For feature enhancement, attention-gated fusion [28] and efficient path aggregation networks [29] improve multi-scale representation, while binocular vision combined with keypoint detection enables non-contact estimation of fish size and mass [30]. In the broader domain of precision livestock farming, computer vision-based body measurement has been extensively investigated for terrestrial animals such as cattle and pigs, with systematic reviews documenting both the technical advances and the persistent challenges of occlusion, posture variation, and breed diversity [31]. However, underwater vision-based fish weight estimation presents additional difficulties—including light attenuation, turbidity, and non-rigid body deformation—that are not adequately addressed by methods developed for terrestrial livestock. Nevertheless, for laterally compressed species such as largemouth bass, three critical gaps remain. First, single-view images cannot capture the three-dimensional morphology necessary for accurate weight prediction, and existing dual-view approaches fuse features only at a shallow post-processing level rather than embedding morphological priors into deep multi-scale feature learning. Second, conventional attention mechanisms lack dedicated suppression of underwater noise from turbidity, debris, and illumination variation. Third, generic pruning strategies risk discarding channels or attention heads critical for morphological representation, and most existing methods have only been validated in the laboratory, lacking robust support for intensive pond deployment.

**Figure 1:**
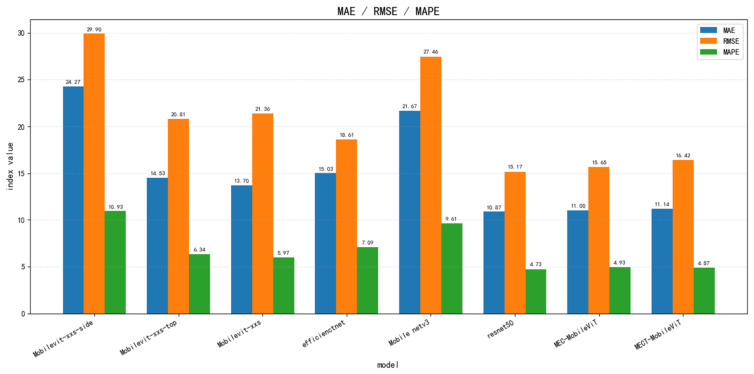
Bar chart comparing the performance of different models in the task of predicting fish weight. The chart shows three key regression metrics, including MAE, MAPE, and RMSE, where smaller values of each metric indicate better model performance.

To address the above industrial demands and research gaps, this paper proposes MECT-MobileViT, a lightweight dual-view weight prediction framework for largemouth bass. The primary novelty lies in the synergistic integration of dual-view feature learning, morphometric prior-guided fusion, anti-interference attention, and multi-granularity pruning, specifically tailored for the laterally compressed body shape of largemouth bass and complex underwater conditions. The main contributions are as follows:

(1) A dual-branch feature extraction architecture is constructed by adapting the MobileViT backbone, independently processing lateral-view and top-view images to capture complementary morphological information, overcoming the single-view prediction bottleneck inherent to laterally compressed fish.

(2) A Morphology-Guided Multi-Scale Fusion (MGMSF) module is designed by integrating deformable convolution with explicit morphometric prior injection, encoding physical measurements (body length, height, and width) and embedding them into multi-scale deep feature fusion, thereby achieving deep coupling between biological prior knowledge and visual features.

(3) An ECA-NL (Efficient Channel Attention with Non-Linear Enhancement) module is formulated by combining instance normalization, GLU gating, and attention threshold filtering with efficient channel attention. The module is designed to improve feature robustness in challenging imaging conditions and to strengthen the representation of key morphological features.

(4) A synergistic lightweight strategy is adopted, combining attention head pruning, dependency-graph-based structured channel pruning, and depthwise separable self-attention replacement, drastically reducing parameter count and computational burden while preserving accuracy, thus providing a viable pathway from accurate model training to edge deployment. The comprehensive performance of MECT-MobileViT is illustrated in Figure 1.

## 2. Materials and Methods

### 2.1. Dataset Description

Precise quantification of morphometric parameters, combined with measured ground-truth body weight to provide reliable supervisory signals for the model, can effectively support research on real-time, non-destructive, and accurate weight prediction of largemouth bass under complex aquaculture conditions.

#### 2.1.1. Data Collection and Scenario Design

The images comprising the dataset were collected in 2025 at the Zongnan Standardized Fish Farming Base in Jishou, Xiangxi, Hunan Province, China, with largemouth bass (*Micropterus salmoides*) as the target species. Thirty healthy individuals covering different growth stages were selected, with body lengths ranging from 20.5 to 26.5 cm and body weights from 150–326 g. It is important to note that all images in the final dataset were extracted from these 30 fish; therefore, the effective biological replication is limited to 30 independent samples. Based on the practical monitoring requirements of the production site, a single standardized dual-view acquisition scenario was designed. To address the potential complexities encountered during fish weight prediction, the constructed dataset incorporated multiple typical scenarios, including images captured under varying illumination conditions and images in which several largemouth bass of different lengths and sizes appeared within the same frame.

**Figure 2:**
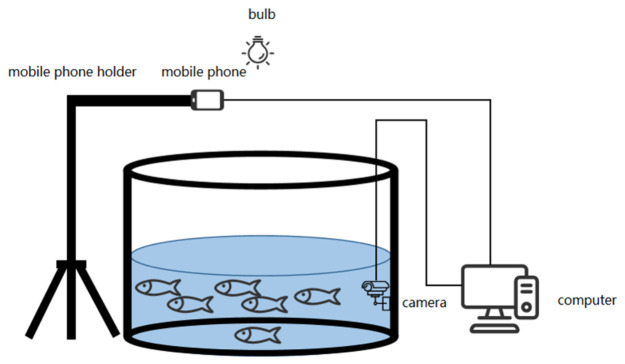
The specific simulated aquaculture environment is illustrated.

A dual-view acquisition system was assembled using a commercial underwater high-definition camera (BENEFITS LEAN FISH BRAND SHOP, Dongguan, China)and a vivo X100S smartphone (vivo Mobile Communications Co., Ltd., Dongguan, China). The underwater camera was fixed to the side wall of the tank to record lateral-view videos, while the vivo X100S was mounted on a custom-designed bracket directly above the tank to capture top-view videos, thereby ensuring that the fields of view of both devices completely covered the fish activity area. The resolutions of the camera and smartphone were set to 1080 p (lateral view) and 720 p (top view), respectively, with a frame rate of 30 fps, to guarantee image clarity and synchronization. The specific imaging environment is illustrated in Figure 2.

The image acquisition was carried out in an indoor controlled tank at the aquaculture base. The tank had a top outer diameter of 0.92 m, a bottom outer diameter of 0.71 m, and a height of 0.7 m, with a water depth of 0.5 m. Water transparency was maintained between 50 and 60 cm to simulate practical aquaculture water conditions and to prevent the loss of fish body features that would result from extremely turbid water. Dual-view videos were recorded separately under two illumination conditions—sufficient daytime illumination and insufficient nighttime illumination—so that different lighting scenarios were covered.

**Figure 3:**
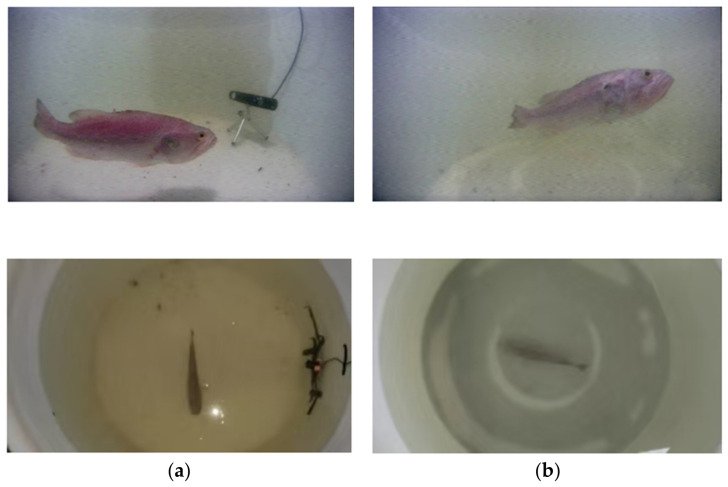
Representative images from the self-collected largemouth bass dataset. (**a**) Lateral and top views of the same individual captured at the same moment under sufficient daytime illumination. (**b**) Lateral and top views of the same individual captured at the same moment under low-light nighttime conditions.

A single fish was introduced into the simulated culture tank. Once steady swimming behavior was established, synchronized recording was triggered. For each fish, 2–3 min of synchronized video was collected, ensuring that various swimming postures, including straight swimming, slight bending, and slow turning, were covered to guarantee postural diversity among the samples. Representative examples of the captured images are shown in Figure 3.

#### 2.1.2. Image Extraction and Naming Conventions

Image extraction from the synchronously recorded lateral-view and top-view videos was performed using Kinovea version 0.9.5. The detailed procedure was as follows:

Video Import and Temporal Alignment: The lateral-view and top-view videos of an individual fish were imported into Kinovea. Precise alignment of the two videos (time difference ≤ 1 frame) was achieved through the built-in timeline calibration function based on the recording start timestamps.

Image Extraction Strategy: A fixed-interval extraction scheme was adopted, whereby one pair of dual-view images was captured every 10 frames (approximately 0.33 s). This ensured that the samples were non-redundant and covered the entire swimming cycle. During extraction, the frame-freezing function of Kinovea was utilized to preferentially select and save frames in which the fish exhibited an extended body posture, no occlusion, and a clearly defined outline.

Naming Conventions: The extracted lateral-view images were named in the format “luyu_side_1080p_fishID_imageNumber.jpg,” and the corresponding label files were named “luyu_side_1080p_fishID_imageNumber.json.” The top-view images were named “luyu_top_720p_fishID_imageNumber.jpg,” with the label files named “luyu_top_720p_fishID_imageNumber.json.” This naming convention ensured a one-to-one correspondence between the dual-view images of the same fish, facilitating subsequent data association and model training.

#### 2.1.3. Ground-Truth Acquisition and Annotation Protocol

Prior to manual measurement, each fish was lightly sedated in a buffered tricaine methanesulfonate (MS-222) solution at a concentration of 100 mg/L to minimize handling stress and prevent injury from struggling. The time out of water for each individual was strictly limited to under 60 s. After measurements, each fish was immediately placed in a dedicated, well-aerated recovery tank and monitored for the return of normal opercular movement and righting reflex before being returned to the original holding tank. The entire sedation and recovery process typically required 5–10 min. Throughout the study, no mortalities or adverse effects were observed. These procedures were conducted in accordance with internationally accepted principles for animal research, including the “3Rs” (Replacement, Reduction, and Refinement), as no institutional animal ethics committee was available for this specific type of research at the time of its execution. The overarching goal of this study—developing a non-contact weight estimation technology—is ethically motivated by its potential to eventually eliminate such stressful physical handling in routine aquaculture practice.

Immediately after image acquisition, physical measurements were taken from each fish to obtain the ground-truth. Body weight was recorded to the nearest gram using an electronic scale, and body length was measured to the nearest millimeter as the straight-line distance from the snout tip to the caudal fin terminus using a measuring board.

To ensure the reliability of the ground-truth data, the electronic scale was calibrated against a standard 200 g weight immediately before each measurement session and exhibited a drift of less than ±1 g. Body weight was recorded once per fish. Because the total out-of-water handling time was strictly limited to less than 60 s, repeated weighings on the same individual were not feasible; we therefore acknowledge that within-individual measurement repeatability was not directly estimated in this study. Body length was measured once to the nearest 1 mm by the same experienced operator using a flat measuring board firmly aligned against the fish’s body. All physical measurements were immediately linked to the corresponding image identifiers to prevent data mismatches.

All measurement data were strictly linked to the corresponding images through unique identifiers. To ensure data quality, a multi-stage quality assurance protocol was established. In the first stage, images exhibiting severe motion blur, localized occlusion covering more than 30% of the body area, or overexposure were rapidly eliminated. In the second stage, the remaining images were meticulously reviewed to ensure that the body contour was clearly defined and the morphological features were complete, and any controversial samples were discarded. As a result, 1172 high-quality images were retained from the original set. However, these images are not independent biological replicates; they constitute repeated video frames from the same 30 fish individuals, captured under varied postures and illumination conditions.

Manual annotation was conducted using the LabelMe tool according to the following rules:

Lateral-view annotation: Four keypoints were annotated—the snout tip (1), the center of the caudal fin (2), the anterior insertion of the dorsal fin (3), and the anterior insertion of the caudal fin (4)—and the entire fish body was enclosed with a bounding box (label name: lu_fish). Body length was calculated as the Euclidean distance between keypoints 1 and 2, and body height was calculated as the perpendicular distance from keypoint 3 to the ventral contour of the fish.

**Figure 4:**
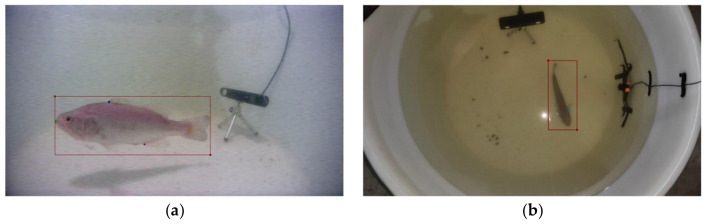
Schematic illustration of the dual-view image annotation. (**a**) Annotation schematic for the lateral view. (**b**) Annotation schematic for the top view.

Top-view annotation: Two keypoints were annotated, with the fish head oriented upward taken as the positive direction—the leftmost point of greatest body width (5) and the rightmost point of greatest body width (6)—and the entire fish body was enclosed with a bounding box (label name: lu_fish). Body width was calculated as the Euclidean distance between keypoints 5 and 6. The specific annotation scheme is illustrated in Figure 4.

**Figure 5:**
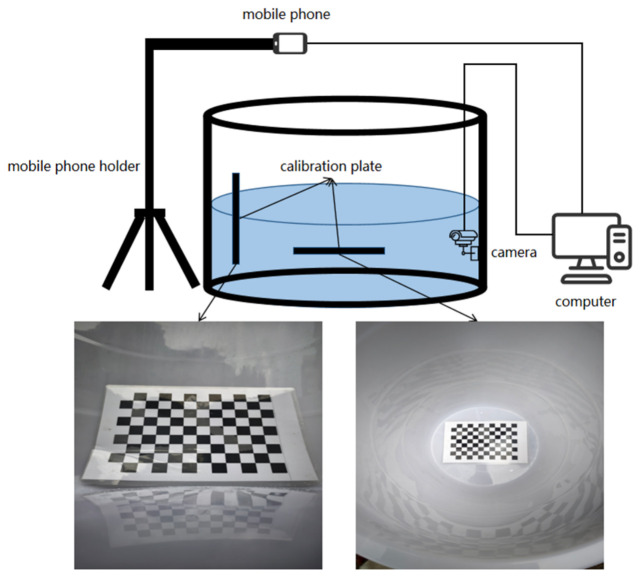
Camera calibration setup for pixel-to-physical-unit conversion.

To convert keypoint distances from pixel units into physically meaningful length units (mm), a dedicated camera calibration was performed for both the lateral-view and top-view cameras. A standard 9 × 12 black-and-white checkerboard with a square size of 20 mm was used as the calibration target. The checkerboard was placed underwater at the depth plane where the fish typically swam, and 25–30 images were captured with each camera at slightly different orientations (Figure 5).

#### 2.1.4. Dataset Partitioning

To rigorously evaluate the generalization capability of the model and to prevent information leakage between the training and validation sets arising from different postural frames of the same fish, a partitioning strategy based on grouping by fish identifier (fish_id) was adopted. The 30 fish were randomly divided into two groups: all images of 24 fish were assigned to the training set, and all images of 6 fish were assigned to the validation set, yielding 936 and 236 images, respectively, corresponding to a ratio of approximately 8:2. It should be noted that this validation set, held out at the individual level, serves as the sole external evaluation set for all model performance assessments in this study; no additional independent test set was withheld. Owing to the random assignment of fish IDs, the validation set weight range (150–260 g) does not fully cover the upper range of the training set (163–326 g), which may limit the assessment of model performance on heavier individuals. To complement the single-split evaluation, a 5-fold fish-level cross-validation was additionally performed for the proposed model (see Section 3.5). The detailed partitioning is presented in Table 1.

#### 2.1.5. Data Preprocessing and Augmentation

To improve model generalization and mitigate overfitting, a systematic data preprocessing and augmentation pipeline was constructed, and differentiated processing was applied to the training set and the validation/test sets.

Preprocessing: All images were uniformly resized to 224 × 224 pixels and normalized using the mean [0.485, 0.456, 0.406] and standard deviation [0.229, 0.224, 0.225] computed from the ImageNet dataset. The body length and body weight labels were standardized, with the parameters fitted solely on the training set, to balance dimensional differences.

Training set augmentation: A compound strategy combining geometric and photometric transformations was adopted, including random horizontal flipping, random rotation, color jittering, and random grayscale conversion. For the dual-view data, the augmentation operations were synchronously performed on the lateral-view and top-view images to maintain view consistency. Table 2 summarizes the detailed pipeline and parameter settings for image data augmentation and preprocessing.

### 2.2. Overall Model Architecture

Pipeline Overview. The complete inference pipeline of MECT-MobileViT proceeds through the following stages:

(1) Input: A synchronized pair of lateral-view and top-view images, both resized to 224 × 224, is fed into the network.

(2) Backbone: A shared-weight MobileViT-XXS dual-branch backbone independently extracts hierarchical feature maps at three scales (shallow, middle, deep) from each view.

(3) Dual-view feature extraction: The lateral branch captures cues for body length and body height; the top-view branch captures body width.

(4) Morphometric prior extraction: The dual-branch backbone simultaneously regresses the keypoints defined in Section 2.1.3—the lateral branch outputs keypoints for body length and body height, and the top-view branch outputs keypoints for body width. From these network-predicted keypoints (rather than any manually measured or externally provided values), body length L, body height H, and body width W are computed on the fly. These geometric parameters are then encoded into a high-dimensional morphometric prior vector via two fully connected layers (Equation (2)).

(5) Feature fusion (MGMSF): The MGMSF module fuses the multi-scale dual-view features, guided by the morphometric priors, to produce a unified, shape-aware representation (Section 2.3).

(6) Feature enhancement (ECA-NL): The ECA-NL module employs instance normalization, gated activation, and threshold filtering to reinforce salient morphological channels and improve robustness under variable underwater conditions (Section 2.4).

(7) Regression output: The enhanced features are passed through a lightweight regression head to predict body weight; the training objective jointly optimizes keypoint localization, bounding box regression, and weight prediction losses (Section 2.5).

(8) Pruning and fine-tuning: After training, a three-stage collaborative pruning strategy—attention head pruning, dependency-graph-based structured channel pruning, and depthwise separable self-attention replacement—compresses the model. A brief fine-tuning phase with a reduced learning rate recovers any minor accuracy loss (Section 2.6).

MobileViT-XXS is recognized as a classic lightweight hybrid vision Transformer in which an optimal balance between feature representation performance and computational efficiency is achieved by integrating the local fine-grained extraction capability of convolutional neural networks with the global context modeling capability of Vision Transformers; consequently, it is adopted as an ideal backbone for vision-based monitoring tasks at the aquaculture edge. Its backbone is alternately composed of shallow convolutional downsampling modules, MobileViT hybrid building blocks, and deep convolutional projection modules. In the early stages, local morphological features such as fish body contours, keypoints, and textures are extracted via depthwise separable convolutions; in the middle stages, global spatial dependencies of the fish body are captured through the multi-head self-attention mechanism of the Transformer; and in the later stages, hierarchical multi-scale feature maps are output, with high feature modeling capability maintained under a very small parameter budget.

**Figure 6:**
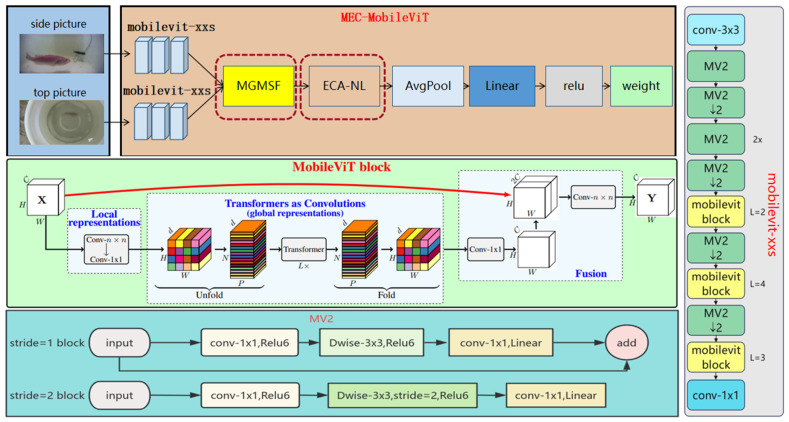
Network architecture of MEC-MobileViT.

For the task of weight prediction in largemouth bass, to address challenges including incomplete morphological representation from a single view, strong underwater noise interference, the need for independent modeling of dual-view features, and the requirement for adaptive fusion of multi-scale features, a series of tailored improvements were made to the MobileViT-XXS backbone. The positions of the key improved modules within the network architecture are indicated by the red dashed boxes in Figure 6, and the improved dual-branch MECT-MobileViT network structure is illustrated in the same figure.

### 2.3. MGMSF: Morphology-Guided Multi-Scale Fusion Module

The MGMSF module receives the multi-scale feature maps from the dual-branch backbone and fuses them under the explicit guidance of morphological priors (body length, height, and width).

**Figure 7:**
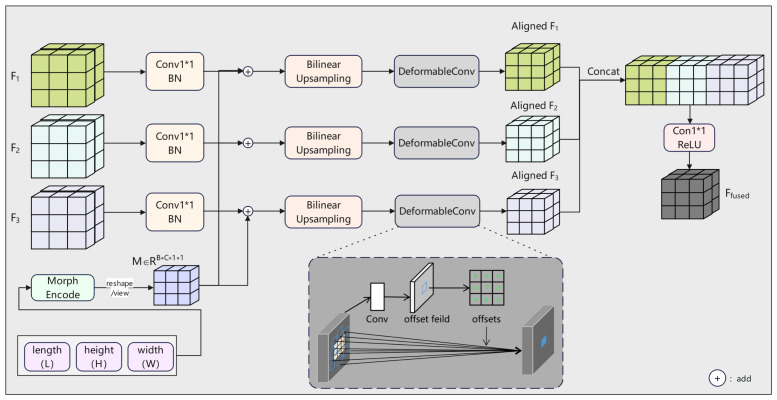
Network architecture of the MGMSF module.

Conventional multi-scale feature fusion modules aggregate shallow details and deep semantic features merely through operations such as upsampling and concatenation, without incorporating the physical prior knowledge of the target, which results in weak correlation between dual-view features, feature alignment deviations caused by fish posture deformation, and low coupling between morphological features and weight regression. To address the core challenges encountered in the weight prediction task for largemouth bass—namely, the absence of physical guidance in dual-view multi-scale feature fusion, poor adaptability to non-rigid deformation of the fish body, and insufficient association between morphological features and body weight—the MGMSF (Morphology-Guided Multi-Scale Fusion) module was designed in this study. The structure of the improved MGMSF morphometry-guided multi-scale fusion module is illustrated in Figure 7.

#### Core Components and Principles of the Module

The MGMSF module achieves deep coupling between physical priors and visual features through an integrated design that encompasses multi-scale projection, morphometric encoding injection, deformation correction, and feature aggregation. The core components and their underlying principles are described below:

(1) Multi-scale projection layer: This layer is composed of three parallel “*1* × *1* convolution + batch normalization (*BN*)” units. The feature maps at three scales output by the dual branches, with channel numbers of *64*, *128*, and *256*, respectively, are uniformly projected to *256* channels, thereby eliminating inter-scale channel discrepancies and providing a compatible basis for subsequent fusion. The mathematical formulation is given in Equation (1):

(1) $$F_{i}^{proj}=BN({Conv}_{1\times 1,256}(F_{i})),i\in \{0,1,2\}$$

In Equation (1), $F_{i}$ denotes the original feature map of the i-th layer, ${Conv}_{1\times 1,256}$ represents a *1* × *1* convolution with *256* output channels, and *BN* denotes batch normalization.

(2) Morphometric feature encoding: The body length *L* and body height *H* predicted by the lateral-view branch, together with the body width *W* predicted by the top-view branch, are concatenated into a three-dimensional vector [*L*,*H*,*W*]. This vector is then passed through two fully connected layers to produce a high-dimensional morphometric prior with a spatial size of *1* × *1*, thereby mapping the physical parameters into the feature space. The mathematical formulation is given in Equation (2):

(2) $$F_{morph}=ReLU({FC}_{1}(V)),F_{sci}=Reshape({FC}_{2}(F_{morph}))\in R^{B\times 256\times 1\times 1}$$

In Equation (2), *V* = [*L*,*H*,*W*] denotes the concatenated morphometric vector; ${FC}_{1}$ and ${FC}_{2}$ are fully connected layers with an intermediate dimension of *128* and an output dimension of *256*, respectively; and the Reshape operation converts the resulting vector into a *1* × *1* spatial tensor.

(3) Morphometric-guided injection: The morphometric prior feature map $F_{sci}$ is element-wise added to each projected feature map, thereby achieving channel-wise bias modulation with morphological priors:

(3) $$F_{i}^{guide}=F_{i}^{proj}⊕F_{sci},\forall i\in \{0,1,2\}$$

As indicated in Equation (3), ⊕ denotes element-wise addition. Since the spatial dimensions of $F_{sci}$ are 1 × 1, it is automatically broadcast by PyTorch to the same height and width as $F_{i}^{proj}$. This operation is equivalent to replicating and tiling a global channel bias, enabling all spatial positions to perceive morphometric information with extremely low computational overhead.

(4) Upsampling alignment: The features at the three scales are unified to the maximum spatial resolution (the dimensions of $F_{0}^{proj}$) through bilinear interpolation:

(4) $$F_{i}^{up}={Upsample}_{H_{max}\times W_{max}}(F_{i}^{guide})$$

As indicated in Equation (4), the Upsample operation employs bilinear interpolation, and ($H_{max},W_{max}$) correspond to the height and width of the first-layer feature map.

(5) Deformable convolution alignment: A *3* × *3* deformable convolution is employed to correct the feature shifts induced by variations in fish posture, thereby improving feature consistency across different postures and accommodating the non-rigid deformation that occurs during the swimming of largemouth bass:

(5) $$F_{i}^{align}={DConv}_{3\times 3,p=1}(F_{i}^{up})$$

As indicated in Equation (5), *DConv* denotes the deformable convolution, the offsets of which are learned end-to-end from the body weight regression loss without requiring additional geometric annotations.

(6) Fusion layer: The aligned feature maps from the three scales are concatenated along the channel dimension (resulting in *256* × *3* = *768* channels) and then compressed to *256* channels through a *1* × *1* convolution. Following *ReLU* activation, the fused feature map is obtained and serves as the input for subsequent body weight regression:

(6) $$F_{fuse}=ReLU({Conv}_{1\times 1,256}(Concat[F_{0}^{proj},F_{1}^{proj},F_{2}^{proj}]))$$

In Equation (6), Concat denotes channel-wise concatenation, and ${Conv}_{1\times 1,256}$ represents a *1* × *1* convolution with *256* output channels followed by *ReLU* activation.

The core innovations of the MGMSF module are reflected in three aspects:

(1) Physical morphometric priors are directly embedded throughout the entire multi-scale feature fusion process, rather than being used merely as inputs to the regression layer, thereby strengthening the association with body weight from the feature encoding stage.

(2) The non-rigid deformation of the fish body is adaptively corrected through deformable convolution, addressing the issue of insufficient feature alignment accuracy present in conventional fusion methods.

(3) Element-wise fusion of morphometric features with multi-scale visual features is adopted to achieve synergistic enhancement of global priors and local details, which significantly outperforms traditional simple concatenation strategies.

### 2.4. ECA-NL Nonlinear Enhanced Attention Module

**Figure 8:**
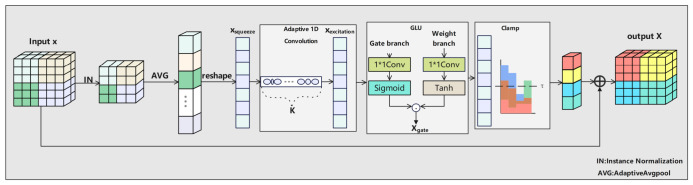
Network architecture of the ECA-NL module.

Following feature fusion, the unified representation is passed to the ECA-NL module, which is designed to enhance feature robustness against common visual interferences in aquaculture settings and to amplify salient morphological features. The architecture of the ECA-NL nonlinear enhanced attention module is illustrated in Figure 8.

#### Core Components and Principles of the Module

(1) Instance normalization layer (InstanceNorm2d): The input feature map $x\in R^{B\times C\times H\times W}$ is normalized to counteract potential feature distribution shifts caused by variable underwater conditions and to stabilize feature responses. The computation is performed as follows:

(7) $$x_{norm}=InstanceNorm2d(x)=\frac{x-\mu }{\sqrt{\sigma^{2}+\epsilon }}$$

As indicated in Equation (7), x represents the input feature map with dimensions B×C×H×W, where B is the batch size (the number of image s input in a single pass), C is the number of channels, and H and W are the spatial height and width, respectively. $x_{norm}$ denotes the normalized output feature map. The term μ is the pixel mean computed per channel, $\sigma^{2}$ is the pixel variance computed per channel, and ϵ is a small constant set to $10^{-5}$ for numerical stability to prevent division by zero.

(2) Adaptive global average pooling: The spatial dimensions are compressed via AdaptiveAvgPool2d(1), yielding a channel descriptor of size [B,C,1,1], which is then reshaped into $x_{squeeze}\in R^{B\times 1\times C}$ to retain global channel-wise information:

(8) $$x_{squeeze}=reshape\left(AdaptiveAvgPool2d\left(x_{norm}\right),(B,1,C)\right)$$

As denoted in Equation (8), AdaptiveAvgPool2d(1) is the adaptive global average pooling operator by which the output size is fixed to 1×1; reshape refers to the dimension reshaping operator; and $x_{squeeze}$ is the channel descriptor vector obtained after spatial compression, with dimensions $R^{B\times 1\times C}$.

(3) Adaptive 1D convolution: The kernel size k is adaptively determined based on the number of channels C to avoid dimensionality reduction loss and to achieve local cross-channel information fusion. The adaptive kernel size is computed as

(9) k=tt+1 if t mod 2=1otherwise, t=|log2C+b|γ

where γ=2 and b=1 are hyperparameters, and the odd constraint on k ensures symmetric padding. The one-dimensional convolution is then performed as

(10) $$x_{excitation}=Conv1d\left(x_{squeeze},\mathrm{kernel}\text{\_}\mathrm{size}=k,padding=\frac{k-1}{2}\right)$$

As specified in Equations (9) and (10), ⌊⋅⌋ denotes the floor operation, and $\log_{2}\left(C\right)$ is the base-2 logarithm of the channel number. The term Conv1d represents the one-dimensional convolution operator, with the adaptively computed kernel size k and symmetric padding of $\frac{k-1}{2}$ ensuring that the input and output dimensions remain unchanged. $x_{squeeze}$ is the channel descriptor vector obtained after pooling and reshaping, and $x_{excitation}$ is the cross-channel fused feature output by the 1D convolution.

(4) GLU gated nonlinear activation: Two separate *1* × *1* convolutional branches are introduced to generate the gating signal and the feature weight, respectively. Adaptive feature selection is achieved through element-wise multiplication, replacing the conventional Sigmoid activation and enhancing the nonlinear representation capacity:

(11) $$\begin{array}{cc} gate=Sigmoid\; & \left(\mathrm{gate}\text{\_}\mathrm{conv}\left(x_{squeeze}\right)\right),\;weight\\ & =Tanh\left(\mathrm{weight}\text{\_}\mathrm{conv}\left(x_{squeeze}\right)\right) \end{array}$$

(12) $$x_{gate}=gate⊙weight$$

As indicated in Equations (11) and (12), gate_conv and weight_conv denote two independent *1* × *1* convolutional layers. The Sigmoid activation function generates gating values within the range of 0 to 1, while the Tanh activation function produces feature weights within the range of −1 to 1. The terms gate and weight represent the gating signal vector and the feature weight vector, respectively; ⊙ denotes element-wise multiplication; and $x_{gate}$ is the output feature following GLU gating.

(5) Attention threshold filtering: A minimum weight threshold of 0.1 is enforced through the clamp operation, by which low-activation channels are filtered out, and the model is guided to focus on key morphological features:

(13) $$x_{threshold}=clamp\left(x_{gate},min=0.1\right)$$

As indicated in Equation (13), clamp is the numerical truncation operator; the setting min=0.1 defines the lower bound for attention weights, such that channels below this threshold are regarded as noise and filtered out; and $x_{threshold}$ denotes the purified attention weights obtained after thresholding.

(6) Residual connection: The enhanced features are added element-wise to the original input to mitigate gradient vanishing and improve training stability:

(14) $$x_{out}=x_{norm}⊙x_{threshold}+x$$

As indicated in Equation (14), $x_{norm}$ denotes the normalized feature map, $x_{threshold}$ represents the filtered attention weights, and x is the original input feature map of the module. The element-wise addition “+” helps alleviate gradient vanishing, and $x_{out}$ is the final enhanced feature output by the ECA-NL module.

Compared with the conventional ECA attention mechanism, the core innovations of the ECA-NL module are as follows: (1) The instance normalization layer preemptively suppresses noise-induced feature distribution shifts. (2) GLU gating activation strengthens the nonlinear capacity for feature selection. (3) The attention threshold mechanism actively filters out invalid noise. (4) The residual connection safeguards gradient propagation. The synergistic combination of these four design elements is intended to improve feature robustness in challenging visual conditions often encountered in aquaculture, such as turbid water and cluttered backgrounds. The specific noise-suppression efficacy remains to be quantified through controlled experiments.

### 2.5. Loss Function and Training Strategy

During model training, multiple objectives—including keypoint localization, bounding box regression, and body weight prediction—are jointly optimized. The total loss function is defined as follows:

(15) $$L_{total}=\lambda_{kp}L_{kp}+\lambda_{box}L_{box}+\lambda_{mass}L_{mass}$$

where $L_{kp}$ denotes the Smooth L1 loss for keypoint coordinates, $L_{box}$ represents the IoU loss for bounding boxes, and $L_{mass}$ is the Huber loss for body weight regression. Based on validation set performance, the loss weights were set to $\lambda_{kp}=0.1$, $\lambda_{box}=0.5$, and $\lambda_{mass}=2.0$, thereby emphasizing the central role of weight prediction. Training was performed using the AdamW optimizer with an initial learning rate of ${1\times 10}^{-4}$, a batch size of 16, and cosine annealing decay to ${1\times 10}^{-6}$ over a total of 200 epochs. The random seed was fixed at 42, and mixed-precision training was adopted to accelerate convergence.

### 2.6. Collaborative Pruning Optimization Strategy

After the initial model training, the three-stage collaborative pruning strategy—positioned as the final step of the overall pipeline—is applied to achieve lightweight deployment while preserving predictive accuracy.

To meet the requirements of lightweight deployment on edge computing terminals, a three-stage collaborative pruning strategy is proposed in this section. The model is compressed sequentially through attention head pruning, dependency graph-based convolutional channel pruning, and depthwise separable self-attention replacement, achieving a significant reduction in parameter count and computational complexity while ensuring that prediction accuracy is preserved.

The computational redundancy of MobileViT is distributed across three levels: attention heads, convolutional channels, and the self-attention operator. Single-granularity pruning cannot comprehensively eliminate such redundancy and may easily damage key morphological features. Therefore, the present strategy follows a progressive sequence of “attention heads → convolutional channels → self-attention computation,” with compression at each level carried out on the premise of preserving the model’s ability to represent fish morphology.

(1) Structured attention head pruning

**Figure 9:**
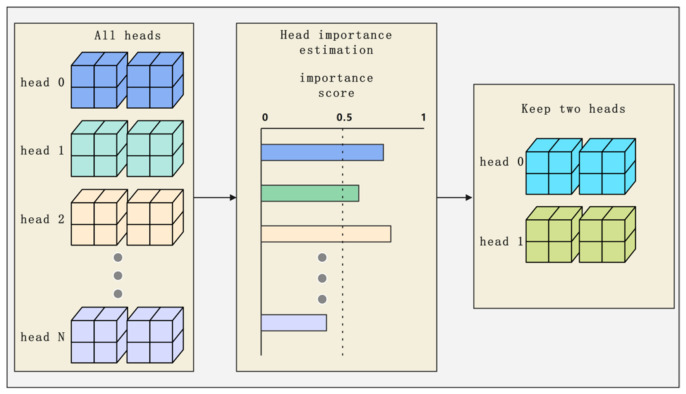
Structured pruning of attention heads.

In multi-head self-attention, some attention heads are irrelevant to the morphological representation of the fish; their outputs focus mainly on background texture or illumination variations. Retaining these heads not only increases computational cost but may also introduce noise. To address this, the Pearson correlation coefficients between the output features of each attention head and the annotated fish keypoints (snout tip, caudal fin, and body width points) are computed as importance scores. Only the two attention heads with the highest scores are retained, and the weights of the QKV projection layers are reconstructed accordingly. This operation directly couples the pruning criterion with the task objective, forcing the attention computation to concentrate on morphologically sensitive regions and suppressing irrelevant semantic branches at the source. The structured pruning of attention heads is illustrated in Figure 9.

(2) Dependency graph-based structured pruning of convolutional channels

After attention head pruning, a substantial number of channels with negligible contributions still remain in the convolutional layers. However, directly removing channels layer by layer would break the parameter dependency relationships between layers, resulting in structural discontinuity. A structured pruning method based on dependency graphs (DepGraph) is introduced. By constructing a parameter dependency graph, channels with cross-layer coupling relationships are grouped into the same parameter groups, ensuring that the network topology remains intact after pruning. During training, group-level L2-norm sparse regularization is imposed to force the weights of redundant channels to decay naturally, and eventually the 20% of channels with the smallest L2 norms are uniformly removed from each convolutional layer. This design achieves channel-level parameter reduction while ensuring safety.

(3) Depthwise separable self-attention replacement

**Figure 10:**
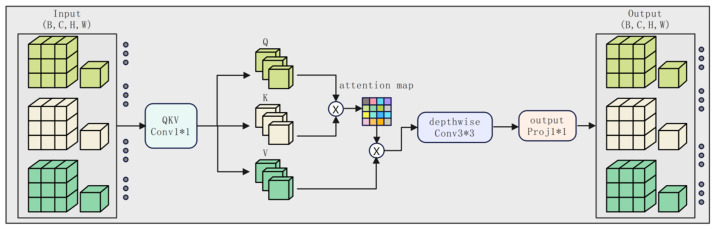
Structure of the depthwise separable self-attention module.

The global spatial interaction of standard self-attention exhibits quadratic complexity with respect to feature map size, making it difficult to meet real-time inference demands. Standard self-attention is therefore replaced with depthwise separable self-attention: a single 1 × 1 convolution is used to simultaneously generate the query, key, and value, thereby reducing the number of linear projection layers; and grouped depthwise convolution (groups = embed_dim) is adopted to replace the global pairwise similarity computation, realizing lightweight per-channel spatial enhancement. During this replacement, the number of attention heads and the embedding dimension are kept unchanged, and only the attention computation unit is modified, thus maximally preserving the previously learned features. The structure of the depthwise separable self-attention module is illustrated in Figure 10.

The three strategies are synergistic and complementary: attention head pruning and channel pruning first reduce parameter redundancy, providing a streamlined structural basis for subsequent operator lightweighting; depthwise separable self-attention replacement then substantially decreases the computational intensity during inference. Through this progressive three-stage compression, both parameter count and computational cost are reduced, while the degradation of critical feature representations—such as morphometric measurements and body contours—that may result from single coarse-grained pruning is avoided. After pruning, the parameter count of the resulting model, MECT-MobileViT, is reduced to 7.34 M, corresponding to a compression of 16.7%, with only minimal fluctuation in accuracy, demonstrating that this strategy achieves an effective balance between lightweight design and high accuracy.

A slight degradation in accuracy is observed after pruning; therefore, fine-tuning with a reduced learning rate is performed on the training set to recover performance. The fine-tuning learning rate is set to one-tenth of the initial learning rate, and training is conducted for 50 epochs. This allows the model, under its lightweight structure, to re-fit the mapping between morphological features and body weight, alleviating the degradation of feature representation and attaining an optimal trade-off between accuracy and model compactness.

## 3. Results

### 3.1. Experimental Environment

All experiments in this study were conducted within a unified hardware and software environment to ensure the reproducibility and comparability of the results. The specific configurations are detailed below.

(1) Hardware platform

The experimental workstation was configured as follows: the central processing unit (CPU) was an AMD Ryzen 7 9700X (8 cores, 16 threads, base frequency 4.7 GHz); 16 GB of DDR5-5600 high-speed memory was installed; the graphics processing unit (GPU) was an NVIDIA GeForce RTX 5070 (16 GB GDDR6X, 7680 CUDA cores); and a 1 TB high-performance NVMe solid-state drive (sequential read speed ≥ 3500 MB/s) was employed for storage, providing high-speed support for data loading and model training.

(2) Software environment

The operating system was Windows 10 Professional (64-bit). Python 3.9.19 was adopted as the programming language. The deep learning framework was built on PyTorch 2.0.1 with CUDA 12.9 enabled for GPU acceleration. Auxiliary libraries, including NumPy 1.26.4, OpenCV 4.9.0, and Scikit-learn 1.4.2, were utilized for data processing, feature computation, and result evaluation.

(3) Basic hyperparameter settings

The basic hyperparameters for model training and optimization were uniformly configured as presented in Table 3. The same hyperparameter settings were applied to all comparative experiments, with only model-structure-related parameters being adjusted, so that interference from hyperparameter differences could be excluded from the experimental results.

### 3.2. Evaluation Metrics

To comprehensively evaluate the performance of the model on the tasks of body length and body weight prediction for largemouth bass, the following four widely used regression evaluation metrics were adopted:

(1) Root Mean Square Error (RMSE): $RMSE=\sqrt{\frac{1}{N}\sum_{i=1}^{N}(y_{i}-{\hat{y}}_{i}{)}^{2}}$. This metric quantifies the dispersion between the predicted values and the true values and is particularly sensitive to larger errors. A smaller RMSE value indicates higher prediction accuracy.

(2) Mean Absolute Error (MAE): $MAE=\frac{1}{N}\sum_{i=1}^{N}|y_{i}-{\hat{y}}_{i}|$. This metric reflects the average absolute magnitude of the prediction errors. It is insensitive to outliers and provides an intuitive representation of the actual level of prediction error.

(3) Mean Absolute Percentage Error (MAPE): $MAPE=\frac{100\%}{N}\sum_{i=1}^{N}\left|\frac{y_{i}-{\hat{y}}_{i}}{y_{i}}\right|$. This metric quantifies the relative error in percentage form, thereby removing the influence of scale and facilitating comparisons across prediction tasks of different magnitudes.

(4) Coefficient of Determination ($R^{2}$): $R^{2}=1-\frac{\sum_{i=1}^{N}(y_{i}-{\hat{y}}_{i}{)}^{2}}{\sum_{i=1}^{N}(y_{i}-\hat{y}{)}^{2}}$. This metric represents the proportion of variance in the target variable that is explained by the model. It ranges from 0 to 1, with values closer to 1 indicating a better goodness-of-fit.

### 3.3. Comparative Experiments and Analysis

To systematically validate the effectiveness of the MEC-MobileViT model, which integrates the Morphology-Guided Multi-Scale Fusion (MGMSF) module and the ECA-NL nonlinear enhanced attention module, a variety of mainstream models covering classical CNNs, hybrid architectures, and Vision Transformers were selected for benchmark testing. All models were trained from scratch on the largemouth bass dataset and evaluated on the held-out validation set (6 fish, 236 images) under the unified experimental environment described in Section 3.1, ensuring fairness. The comparative experimental results of different models are presented in Table 4.

As can be observed from Table 4, the single-branch models that relied solely on the top view or the side view (MobileViT-XXS-top and MobileViT-XXS-side) yielded markedly higher errors, with $R^{2}$ values of only 0.7214 and 0.4247, respectively, revealing the fundamental limitation that a single view is insufficient to capture the laterally compressed body shape of largemouth bass. Although the standard MobileViT-XXS, which receives dual-view input simultaneously, raised the $R^{2}$ to 0.7615, its shallow fusion strategy based merely on channel concatenation failed to fully exploit the deep coupling between morphometric parameters and visual features; consequently, a considerable gap in accuracy remained relative to the proposed model.

The proposed MEC-MobileViT, with only 8.81 M parameters, achieved the highest $R^{2}$ (0.8424) and the second-lowest RMSE (15.6546). Its overall predictive capability was comparable to that of ResNet50 ($R^{2}=0$.8085, RMSE = 15.1691), which possesses nearly eight times the number of parameters, and it exhibited a clear advantage in the coefficient of determination. Although MEC-MobileViT was slightly inferior to ResNet50 in terms of MAE and MAPE, the differences were marginal (MAE differed by only 0.13, MAPE by only 0.2%), whereas the model size and computational overhead were compressed by an order of magnitude, indicating that a superior balance between accuracy and lightweight design was attained. This advantage is mainly attributed to two core innovations. First, the MGMSF module explicitly encodes physical morphometric priors—body length, body height, and body width—and embeds them into the multi-scale feature fusion process, thereby guiding the network at the feature level toward morphological representations that are strongly correlated with body weight and significantly strengthening the mapping between visual features and weight. Second, the ECA-NL module suppresses feature distribution shifts caused by the underwater environment through instance normalization and further filters out background noise such as turbidity and debris via GLU gating and attention thresholding. As a result, sensitivity to critical features including fish body contours and morphometric keypoints is consistently maintained in complex aquaculture water. In contrast, none of the conventional CNN or Transformer baselines incorporated such physically guided and anti-interference attention mechanisms, which consequently limited their predictive capability.

After pruning, the parameter count of MECT-MobileViT was further reduced to 7.34 M, corresponding to a compression ratio of 16.7%. Under this lightweight structure, the $R^{2}$ remained at 0.8266, the RMSE increased only slightly to 16.4201, and the MAPE was further reduced to 4.87%, even outperforming some unpruned baseline models. This remarkable balance between accuracy and efficiency can be attributed to the effectiveness of the three-stage collaborative pruning strategy: attention head pruning retains only the heads most responsive to key morphological features of the fish; structured channel pruning safely removes redundant channels in cross-layer dependency relationships; and depthwise separable self-attention replacement substantially reduces the computational density of the remaining attention modules. Operating in a progressive and synergistic manner, the three stages not only eliminate invalid parameters but also effectively serve as structural regularization, enabling the lightweight model to converge to a feature space with stronger discriminative power after fine-tuning. Overall, the proposed model achieves an optimal balance between high accuracy and lightweight design. The pruned version, with only 7.34 M parameters and 1.45 GFLOPs, demonstrates a promising lightweight design that may facilitate potential edge deployment, although actual validation on embedded edge hardware remains to be conducted, providing reliable support for real-time, non-contact estimation of largemouth bass body weight.

### 3.4. Ablation Study and Analysis

To systematically evaluate the independent and synergistic effects of the proposed MGMSF morphometry-guided fusion module, the ECA-NL attention module, and the CAT three-stage collaborative pruning strategy, five ablation experiments were designed with the original MobileViT-XXS serving as the baseline network. All experiments were conducted under identical training environments and parameter settings, and the results are presented in Table 5.

The experimental results demonstrate that when only the MGMSF morphometry-guided fusion module was introduced, the weight prediction performance of the MobileViT-XXS model was substantially improved: the $R^{2}$ value increased from 0.7615 to 0.8272, representing an improvement of 8.63%; the RMSE decreased from 18.7820 to 16.3915, a reduction of 12.73%; the MAE decreased from 12.3930 to 11.6997; and the MAPE decreased from 5.50% to 5.29%. These outcomes indicate that, through its morphometry-guided dual-view feature fusion design, the MGMSF module effectively exploited the synergistic information of lateral-view and top-view morphological features of largemouth bass, strengthened the mapping between morphological features and body weight, and consequently enhanced the accuracy and reliability of model predictions.

When only the ECA-NL attention module was introduced, performance improvements were also obtained, though the gains were relatively moderate. The $R^{2}$ increased from 0.7615 to 0.7790, the RMSE decreased from 18.7820 to 18.5354, and the MAPE decreased from 5.50% to 5.15%. This suggests that the module plays a positive role in reinforcing core morphological features and improving feature robustness under variable imaging conditions, thereby contributing to an enhanced anti-interference capability.

Furthermore, when both the MGMSF and ECA-NL modules were introduced simultaneously, the model performance exhibited a superimposed improvement: the $R^{2}$ further increased to 0.8424, the RMSE decreased to 15.6546, the MAE decreased to 11.0009, and the MAPE decreased to 4.93%. This fully reflects the synergistic effect of the two modules—while the MGMSF module is responsible for fusing dual-view features and exploiting the relationship between morphometric measurements and body weight, the ECA-NL module is responsible for strengthening core features and suppressing noise. Together, they effectively resolved the problems of insufficient feature extraction and weak anti-interference capability present in the baseline model.

After the CAT three-stage collaborative pruning strategy was further introduced on the basis of the two-module synergy, the number of model parameters was significantly compressed from 8.81 M to 7.34 M, achieving a compression ratio of 16.7%. Meanwhile, the performance exhibited only slight fluctuations: the $R^{2}$ decreased marginally from 0.8424 to 0.8266, the RMSE and MAE increased slightly, and the MAPE even decreased to 4.87%. This indicates that the pruning strategy was able to effectively remove redundant parameters without compromising the core feature representation capacity of the model, thereby achieving a lightweight design that makes the model more suitable for deployment on edge devices in aquaculture.

### 3.5. Fish-Level Cross-Validation

To address the potential bias introduced by the original random fish-ID split—specifically, the absence of heavier individuals (260–326 g) in the validation set—a 5-fold fish-level cross-validation was conducted for the proposed MECT-MobileViT model. The 30 largemouth bass were first stratified by body weight to ensure a uniform weight distribution, then randomly partitioned into five folds, each containing 6 individual fish. In each of the five iterations, four folds (24 fish) were used for training, and the remaining one fold (6 fish) was held out for validation. All training hyperparameters were kept identical to those described in Section 3.1, including the input resolution, optimizer, loss function, and number of training epochs. The mean and standard deviation of the evaluation metrics across the five folds are reported in Table 6.

As shown in Table 6, the model achieved a mean R^2^ of 0.8186 with a small standard deviation of 0.0122, and a mean RMSE of 16.89 g. These values are highly consistent with the results obtained on the original single validation split (R^2^ = 0.8266, RMSE = 16.42 g, as reported in Table 4), confirming that the model’s prediction accuracy is stable across different subsets of fish individuals. The minimal inter-fold variation further demonstrates that MECT-MobileViT is robust to variations in individual size and body weight distribution, including those heavier individuals that were missing from the original validation set. These findings reinforce the conclusion that the model possesses satisfactory generalization capability for weight prediction across the full range of fish sizes encountered in this study.

### 3.6. Visualization Analysis

A series of visualization analyses were performed to more intuitively demonstrate the stability of the training process of the proposed MEC-MobileViT model and the superior performance of its pruned version, MECT-MobileViT. These analyses encompassed the evolution of the loss function during training and the agreement between the predicted values and the ground-truth values of the final model on the test set.

#### 3.6.1. Training Process Analysis

**Figure 11:**
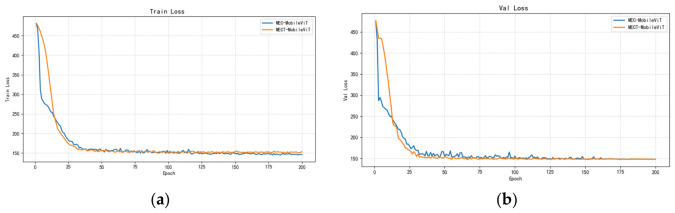
Comparison of training metrics between the MEC-MobileViT and MECT-MobileViT models. The changes in training metrics over the entire 200 training epochs are illustrated. (**a**) Train-loss comparison curve. (**b**) Validation-loss comparison curve.

The training metric curves recorded for the MEC-MobileViT and MECT-MobileViT models over 200 training epochs are presented in Figure 11, where two key trajectories—total training loss and total validation loss—are shown. Several observations can be drawn from the figure.

Stable convergence: Both the unpruned MEC-MobileViT model (blue) and the pruned MECT-MobileViT model (orange) exhibited smooth and efficient convergence trends. During the initial phase (0–25 epochs), the error values declined rapidly from very high initial levels, indicating that the core mapping patterns between the dual-view morphological features of largemouth bass and body weight were efficiently captured within a short period. In the later phase (25–200 epochs), the error curves decreased steadily and tended to stabilize, ultimately converging to extremely low error levels. This behavior validates the effectiveness of the MGMSF morphometry-guided fusion and ECA-NL noise suppression mechanisms and confirms that the pruning operation introduced no training delays, thereby ensuring the overall stability and efficiency of the training process.

Generalization capability: The training and validation losses of MEC-MobileViT and MECT-MobileViT maintained a highly consistent decreasing trend throughout the entire training process, without any rebound or substantial deviation being observed in the validation loss. This indicates that the three-stage collaborative pruning strategy did not impair the feature representation capacity of the model; rather, the risk of overfitting arising from redundant parameters was eliminated, which significantly enhanced the generalization ability and robustness of the model, and ensured that high-accuracy weight prediction can be retained on complex images from real aquaculture scenarios.

Synergistic optimization of the pruning mechanism: The error descent trajectory of the pruned MECT-MobileViT model almost completely overlapped with that of the unpruned model, with a further advantage of smaller fluctuation amplitudes. This demonstrates that the synergistic mechanism combining attention head pruning, structured channel pruning, and depthwise separable attention replacement achieved the ideal effect of “removing redundancy while preserving the core” during model compression. Non-essential parameters and ineffective computations were effectively eliminated by the pruning process, without compromising the model’s ability to learn the core morphological features of the fish. As a result, the lightweight model was driven to converge to a more discriminative feature space, and a synergistic optimum between accuracy and compactness was attained.

#### 3.6.2. Prediction Performance Visualization

**Figure 12:**
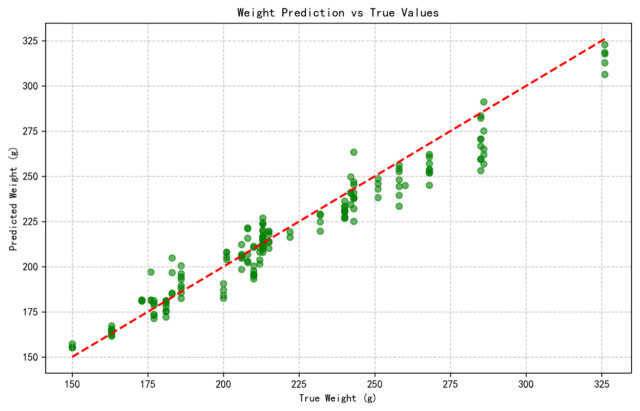
Visualization of the performance of the MECT-MobileViT model on the body weight prediction task (The red dashed line denotes the ideal y = x reference line; green dots represent test samples).

To intuitively evaluate the prediction accuracy of the pruned model, MECT-MobileViT, a scatter plot of the predicted body weight values against the ground- truth values on the validation set is presented in Figure 11. The horizontal and vertical axes represent the ground-truth and predicted values, respectively, and the black dashed line indicates the ideal fit reference line y=x. As can be observed from Figure 12, the data points are closely distributed along the y=x reference line, exhibiting a pronounced linear trend. A coefficient of determination of 0.8266 is achieved, which intuitively verifies the high degree of consistency between the predicted and ground-truth values.

#### 3.6.3. Quantitative Empirical Analysis of the Limitations of Single-View Prediction

To reveal, at the data level, the intrinsic reasons why a single view or a single morphological parameter cannot reliably predict the body weight of largemouth bass, a derived parameter with explicit biological significance, x=body weight/body length, was constructed based on the 30 samples in the present dataset. This parameter can be interpreted as the “linear density” or a simplified “condition factor” of the fish, essentially coupling the information on body height and body width that cannot be captured by body length alone. For a given body length, a higher xx value indicates a plumper fish, i.e., greater body height and/or body width. The results are presented in Table 7.

Based on the analysis of Table 7, three progressive conclusions were drawn.

First, the variation in condition factor disrupts the single-valued mapping from body length to body weight. Although body lengths were concentrated in the range of 20.5–26.5 cm, the coefficient of variation of xx reached as high as 14.1%, with extreme values differing by nearly 70%. The same body length could correspond to substantially different body weights: two individuals with a body length of 22.0 cm had body weights of 173 g and 210 g, a difference of 21.4%; individuals with a body length around 23.5 cm exhibited a body weight span of 201–258 g, with a maximum difference of 28.4%. It is thus evident that treating body weight as a univariate function of body length inevitably neglects the condition factor variation determined by body height and body width, constituting a source of systematic prediction bias.

Second, the error in single-view prediction arises from structural deficiency rather than random perturbation. When body weight was predicted from body length alone, the residuals were strongly positively correlated with the xx values: body weight was underestimated for individuals with high xx and overestimated for those with low xx. This effect is fully consistent with the results of the single-view experiments—the $R^{2}$ was only 0.4247 when only the lateral view (which contains body height) was used, and 0.7214 when only the top view (which contains body width) was used, both far lower than the 0.8424 achieved after dual-view fusion. The systematic unidirectionality of the error directly reflects the missing dimension of body width or body height.

Third, the above mechanism provides theoretical necessity for the dual-branch architecture and the MGMSF module proposed in this work. Single-view projection compresses the three-dimensional morphology into two dimensions, inevitably losing the variation in condition factor. It was precisely to reconstruct this variation in the feature space that the lateral-view and top-view branches were designed to separately extract body height and body width information, and the MGMSF module was employed to deeply couple the explicit morphometric priors (body length, body height, and body width) with multi-scale visual features. Therefore, this analysis not only explains, from the perspective of the data generation mechanism, the fundamental reason why the proposed method outperforms single-view and shallow fusion methods, but also provides a methodological reference for visual weight estimation of laterally compressed fish species.

#### 3.6.4. Attention Map Visualization and Mechanistic Analysis

**Figure 13:**
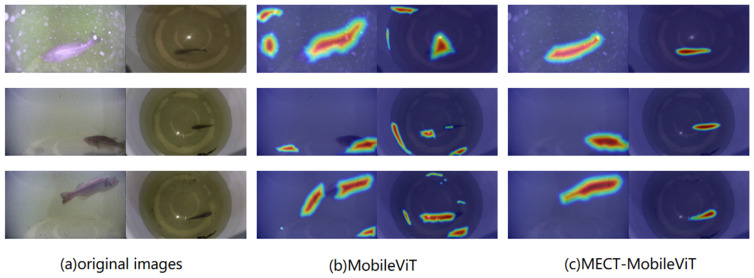
Comparison of Model Heatmap Results (The color gradient from blue to red denotes the level of feature activation; red indicates high-attention regions, and blue indicates low-attention regions).

To complement the quantitative evidence and directly verify whether the proposed MGMSF and ECA-NL modules enhance morphological feature extraction and suppress background noise in practice, a Grad-CAM [32] attention-map analysis was conducted. Representative lateral-view and top-view images from the validation set were selected, deliberately including scenes with turbid water, suspended debris, and non-uniform illumination so as to challenge the models with realistic underwater interference. The MobileViT and the final pruned MECT-MobileViT were compared under identical input conditions (Figure 13).

The contrast between the two models is systematic and revealing:

MobileViT produces widely scattered activation patterns. High-response regions are found not only on partial fish contours but also intensively on water-surface reflections, tank-bottom shadows, and floating particles. This indicates that the baseline model struggles to disentangle morphological cues from environmental noise, and its weight predictions are likely influenced by irrelevant visual textures.

MECT-MobileViT, in contrast, generates tightly focused heatmaps. The highlighted areas are sharply confined to key morphological structures that are functionally linked to body weight. Most strikingly, the activation on background water, debris, and reflections is almost entirely suppressed, even in the images containing suspended particles or low-light conditions. The model’s attention remains robustly locked onto the fish’s shape.

Taken together, the attention-map evidence confirms that MGMSF and ECA-NL do not merely offer abstract performance gains but fulfil their designed roles in a visually interpretable manner: MGMSF steers the network’s focus towards shape-relevant regions, and ECA-NL attenuates the visual clutter inherent to aquaculture environments.

## 4. Discussion

In this study, a lightweight prediction framework, MECT-MobileViT, was proposed, demonstrating the feasibility of non-contact visual prediction of body weight in largemouth bass under experimental conditions, and offering a promising basis for future engineering deployment in smart aquaculture. In this chapter, a systematic discussion is presented including the aspects of model performance mechanisms, comparison with related studies, industrial application value, research limitations and future directions, and theoretical and methodological contributions. The scientific significance, innovative value, and potential impact of the research are comprehensively elucidated.

### 4.1. Interpretation of Results

The significant improvement in model performance achieved in this study is attributed to the deep synergy and mechanistic coupling of multiple modules in the feature space, rather than the linear superposition of individual techniques. A closed-loop mechanism encompassing “feature generation → feature enhancement → noise suppression” was formed by the MGMSF morphometry-guided multi-scale fusion module and the ECA-NL nonlinear enhanced attention module, thereby fundamentally elevating the quality of feature representation and the accuracy of weight regression. Within the MGMSF module, biophysical priors—body length, body height, and body width—were encoded as channel biases and explicitly injected into the multi-scale feature fusion pipeline, enabling the network to concentrate on morphological structures highly correlated with body weight at the feature encoding stage and effectively alleviating the problem, prevalent in conventional methods, of a deep disconnect between physical priors and visual representations. The ECA-NL module is designed with instance normalization, GLU gating, and threshold filtering mechanisms that theoretically counteract feature distribution shifts and suppress low-activation channels, thereby enhancing robustness to visual interference commonly encountered in aquaculture environments. The empirical results indicate improved overall feature robustness; however, the specific contribution of these mechanisms to suppressing turbidity, debris, or illumination noise has not been directly verified through controlled experiments. As demonstrated by the ablation experiments, the introduction of MGMSF alone raised the model’s $R^{2}$ to 0.8272, and the further addition of ECA-NL elevated it to 0.8424 while the MAPE decreased from 5.29% to 4.93%, confirming that physical prior guidance and noise suppression form a positive feedback loop: purified features improve the accuracy of prior guidance, and the strengthened morphological representations further enhance the signal-to-noise ratio of critical channels.

At the structural optimization level, a synergistic balance between efficient redundancy elimination and performance preservation was achieved by the three-stage collaborative pruning strategy. Effective attention heads were selected based on their correlation with key morphological regions of the fish, suppressing noisy semantic branches at the source; redundant channels across layers were safely removed based on dependency graphs, ensuring the integrity of the network topology; and the computational complexity was substantially reduced while the feature representation capacity was maintained through depthwise separable self-attention replacement. After the progressive implementation of these three stages, the model parameter count was compressed by 16.7%, the $R^{2}$ decreased by only 0.0158, and the MAPE was further reduced to 4.87%, indicating that structural sparsification can produce an implicit regularization effect, enabling the model to converge to a superior solution within a more compact parameter space. The above results suggest that lightweight vision systems targeting specific biological morphologies should be co-designed from three dimensions—feature learning, anti-interference capability, and structural compression—and that improvements limited to a single dimension are unlikely to overcome the overall performance bottleneck.

### 4.2. Comparison with Existing Literature

Compared with existing studies, mechanistic innovations and performance breakthroughs were achieved by MECT-MobileViT in three core dimensions: deep dual-view fusion, robust underwater noise suppression, and collaborative lightweight compression. At the level of dual-view feature utilization, the biomass estimation system proposed by Zhang et al. [27] depended on single-view input, and critical three-dimensional information such as body width was consequently absent. The FishKP-YOLOv11 model presented by Chen et al. [29] employed binocular vision, yet only post-processing-level fusion of keypoints was realized, without deep interaction between visual features and physical priors. In contrast, within the MGMSF module of the present study, morphological priors were embedded in the form of channel biases throughout the entire feature fusion process. A closer morphology–weight mapping relationship was thereby constructed under the guidance of prior knowledge, and the limitations of traditional fusion were overcome at the paradigm level. With regard to underwater noise suppression, it was identified by Saleh et al. [32] that water turbidity and uneven illumination constitute the primary factors responsible for the insufficient generalization ability of underwater vision models. Conventional attention mechanisms predominantly rely on Sigmoid weighting, through which noise channels cannot be completely blocked. In the ECA-NL module of this work, GLU gating and threshold filtering are employed to selectively emphasize informative channels, which contributes to more reliable feature extraction under challenging visual conditions. The extent to which this corresponds to targeted noise removal requires further controlled investigation. In terms of lightweight design, single-granularity compression strategies were adopted by both ECR-MobileNet proposed by Peng et al. [25] and the pruned YOLOv7 proposed by Liu et al. [26], which could easily result in the loss of critical features. By contrast, the three-stage collaborative pruning strategy in this work eliminated redundancy at multiple levels—attention heads, convolutional channels, and the attention computation unit—so that morphological features were maximally preserved while the model was compressed, and an optimal balance between accuracy and efficiency was attained.

### 4.3. Significance for Aquaculture

An accurate, low-stress visual weight monitoring solution with strong potential for edge deployment is provided by MECT-MobileViT, which holds considerable industrial application value. Traditional manual capture and weighing is characterized by low efficiency, strong stress induction, and data discreteness, which can easily lead to growth suppression, physical injury, infection, and even mortality in fish, and is therefore insufficient to support precision farming management. In this study, non-contact weight estimation was realized based on dual-view visual perception, whereby the stress risk induced by physical disturbance was completely avoided and the continuity, accuracy, and stability of growth parameter monitoring were improved. Supported by the accurate predictions of the model, dynamic matching of feed amounts and precise feeding decisions can be implemented, contributing to reductions in the feed conversion ratio and residual feed pollution, and enhancing the input–output ratio of farming operations. Continuous body weight data can be directly utilized for growth trend assessment, stocking density optimization, grading and classification, and yield estimation, thereby providing quantitative support for production scheduling in large-scale farming. The non-invasive monitoring mode is consistent with green and healthy aquaculture standards and helps to maintain the stability of the culture water environment and the physiological state of fish stocks. Furthermore, after three-stage collaborative pruning, the model parameter count is only 7.34 M, making it feasible for deployment on embedded edge devices at the farming site, substantially reducing the hardware threshold for practical adoption. Consequently, the hardware threshold and large-scale application cost of intelligent monitoring technology are substantially reduced, and a lightweight, highly practical technical pathway for the intelligent upgrading of industrial farming of largemouth bass is offered.

### 4.4. Limitations

Although significant progress has been achieved in non-contact weight prediction and model lightweighting for largemouth bass in this study, the following limitations remain. First, and most critically, the biological sample size of this study is limited to 30 largemouth bass individuals, all sourced from a single farming location. Although 1172 images were extracted and used for training and validation, these images are repeated frames from the same 30 individuals rather than biologically independent samples. This introduces two interrelated risks: (1) the limited inter-individual variation in body shape, pigmentation patterns, and scale texture may not adequately represent the population-level morphological diversity of largemouth bass across different genetic stocks and farming conditions; (2) the model may inadvertently learn individual-specific visual cues—such as unique fin deformities, scar patterns, or pigmentation idiosyncrasies—rather than generalizable morphology-to-weight mappings, potentially leading to inflated performance metrics on the current validation set that do not transfer to unseen individuals from other populations. Future studies must substantially expand the number of independent biological replicates and incorporate multi-site, multi-strain sampling to rigorously validate population-level generalization. Second, the feature extraction stability of the ECA-NL module under extreme conditions—such as very low illumination, high turbidity, and rapid swimming-induced motion blur—has not been thoroughly examined and requires further improvement. Third, although the dataset incorporates two natural illumination levels (daytime sufficient light and nighttime low-light) and the water was maintained at practical aquaculture turbidity (transparency 50–60 cm), the anti-interference capability of the ECA-NL module has so far been evaluated only indirectly through overall prediction metrics. The binary light condition alone cannot isolate the effect of illumination from other covarying factors, and no factorial control of turbidity, suspended debris concentration, or illumination intensity was implemented. Consequently, the current results cannot attribute performance gains to the suppression of any single environmental factor; a direct causal link between the ECA-NL components and specific noise suppression remains to be established. Future work should include systematic, graded noise injection experiments (e.g., clay suspensions to control turbidity, controlled particulate debris, and adjustable illuminance) to isolate and quantify the module’s noise-filtering contribution under each factor. Fourth, a slight degradation in accuracy accompanies the lightweighting achieved by the pruned MECT-MobileViT; this trade-off effect is more pronounced in weight prediction for small-sized individuals, and further optimization should be conducted in line with practical farming requirements. Furthermore, because only a single held-out validation set was used for model evaluation without an additional independent test set collected from entirely separate farming conditions, the reported performance metrics may reflect a degree of optimistic bias relative to true field deployment. Additionally, no actual on-site deployment on embedded edge devices has yet been performed; all inference was executed on a laboratory GPU workstation. Therefore, the real-world inference latency, power consumption, and robustness under continuous operation on farm-grade hardware remain unverified. Future work should incorporate a truly independent external test set from different farms, water conditions, and fish batches, as well as hardware-in-the-loop deployment tests on representative edge devices, to rigorously benchmark generalization and field deployability. Fifth, while the newly added 5-fold fish-level cross-validation (Section 3.5) provides mean ± SD metrics that serve as confidence intervals and confirm stable generalization across different fish subsets, several diagnostic analyses recommended for a full clinical-level validation remain to be conducted. Specifically, Bland–Altman analysis for systematic bias quantification, residual distribution modeling, prediction error breakdown by body weight strata, and systematic failure case taxonomy would provide finer-grained insights into error characteristics and model limitations. We consider these analyses to be most informative and statistically meaningful when performed on a larger, multi-site, multi-species dataset where the sample size supports robust subgroup comparisons and the diversity of conditions justifies a comprehensive diagnostic framework. In the present proof-of-concept study with 30 fish from a single site, we have prioritized cross-validation as the most direct and widely accepted approach to address the generalization concern, and we defer the full suite of diagnostic evaluations to our planned multi-center validation study. Sixth, the morphometric priors (body length, body height, and body width) that guide the MGMSF module are derived from keypoints predicted by the dual-branch backbone rather than from externally measured ground-truth values. Consequently, the quality of the morphometric guidance is inherently dependent on the accuracy of keypoint localization. Systematic keypoint prediction errors—especially under challenging conditions such as partial occlusion, motion blur, or extreme turbidity—may propagate into the prior encoding and degrade the fidelity of the multi-scale feature fusion, ultimately affecting weight prediction accuracy. Future work should investigate the sensitivity of the overall prediction to keypoint errors and explore robust alternatives, such as incorporating uncertainty-aware prior encoding or decoupling the morphometric regression from the weight prediction pathway.

### 4.5. Future Research Directions

Based on the foundation established in this study, future research can be extended in three directions. First, a large-scale dataset covering multiple geographic regions, culture modes, scenarios, and fish species should be constructed, incorporating samples of varying body sizes, water qualities, lighting conditions, and postures, so that the cross-species generalization ability of the model to closely related percichthyid species can be verified. Critically, this expanded dataset will enable the comprehensive suite of diagnostic validations that are currently deferred due to limited biological replication—including Bland–Altman analysis for systematic bias assessment, residual distribution characterization, prediction error stratification by fish size and condition, and systematic failure case taxonomy across diverse environmental conditions. These analyses, combined with formal statistical hypothesis testing between competing models, will provide the clinical-level evidence required for translational deployment in commercial aquaculture settings. Second, the synergistic mechanism between the MGMSF and ECA-NL modules can be optimized by incorporating neural architecture search (NAS), and hybrid CNN–Transformer architectures can be introduced to improve feature extraction efficiency; the three-stage collaborative pruning strategy can be further refined to achieve a more optimal balance between accuracy and model compactness. Third, the adaptability of the model to complex scenes can be enhanced by using generative adversarial networks to generate samples of extreme environments and by integrating self-supervised learning to improve underwater feature robustness; multi-modal fusion of visual features with environmental parameters such as water quality and water temperature can be explored to further elevate prediction accuracy and engineering practicability. Additionally, building on the existing day/night illumination contrast already present in our dataset, future work will establish a factorial experimental design in which water turbidity (e.g., by adding standardized clay suspensions), suspended debris concentration, and illumination intensity are independently varied at multiple levels. Under such controlled conditions, the pruned MECT-MobileViT model will be evaluated to partition prediction error variance and directly quantify the unique contribution of the ECA-NL module to suppressing each type of environmental disturbance. This will provide the direct causal evidence needed to validate its design rationale.

### 4.6. Theoretical and Methodological Contributions

Three theoretical and methodological contributions to the fields of deep learning and aquatic machine vision are made by this study. First, to address the challenge of heterogeneous fusion between dual-view morphological features and physical priors, an MGMSF morphology-guided multi-scale fusion mechanism was proposed, demonstrating how explicit morphometric priors can be effectively embedded into deep feature fusion for vision-based fish weight regression, offering a reusable integration paradigm. Second, targeting the task of visual perception under low signal-to-noise-ratio underwater conditions, an ECA-NL nonlinear enhanced attention module was constructed by synergistically combining established techniques (instance normalization, GLU gating, and threshold filtering), providing an effective anti-interference solution for lightweight visual modeling in complex aquatic environments. Third, to mitigate the parameter and computational redundancy of lightweight Transformers, a three-stage synergistic compression strategy—combining attention head pruning, structured channel pruning, and depthwise separable self-attention replacement—was constructed. It was demonstrated that such multi-granularity collaborative pruning can achieve efficient model simplification while preserving task-critical feature representations, thereby enriching the empirical framework for structured compression of lightweight CNN–Transformer hybrids.

In summary, through the integrated design of multi-scale feature coupling, anti-interference attention enhancement, and multi-level collaborative pruning, a visual weight estimation model for largemouth bass that balances prediction accuracy with edge deployment efficiency was constructed in this study. MECT-MobileViT not only provides an engineering-deployable solution for stress-free growth monitoring in intensive farming of largemouth bass, but also offers a referable technical pathway for the deployment of CNN–Transformer hybrid architectures in aquatic edge visual perception terminals. Through continuous iteration of the fusion mechanism and compression strategy, this framework can be extended to the morphological parameter monitoring of additional economically important fish species, providing theoretical support and technical assurance for the high-quality development of smart aquaculture toward precision, lightweighting, and large-scale application.

## 5. Conclusions

To meet the core engineering demands of non-contact, accurate, low-stress, real-time prediction of live body weight in intensive largemouth bass aquaculture, this study focused on three bottlenecks—insufficient dual-view feature fusion, pronounced underwater noise interference, and the difficulty of reconciling accuracy with deployment efficiency in lightweight models—and a lightweight prediction framework, MECT-MobileViT, was constructed and systematically validated. The main conclusions and contributions are summarized as follows.

(1) A lightweight framework that achieves competitive accuracy ($R^{2}$ = 0.8424 before pruning, $R^{2}$ = 0.8266 after pruning) tailored for weight estimation of largemouth bass was proposed. MobileViT-XXS was adopted as the backbone, and the MGMSF morphometry-guided multi-scale fusion module together with the ECA-NL nonlinear enhanced attention module were integrated into an end-to-end weight prediction model. The unpruned model achieved an RMSE of 15.6546 and an $R^{2}$ of 0.8424; after pruning, the parameter count was reduced to 7.34 M, the RMSE was 16.4201, and the $R^{2}$ was maintained at 0.8266, corresponding to an accuracy loss of less than 2%. The comprehensive performance significantly surpassed mainstream baselines including ViT, the ResNet series, EfficientNet, and the original MobileViT.

(2) A modeling paradigm that deeply couples dual-view visual features with morphological priors was established. In the MGMSF module, physical priors—body length, body height, and body width—were embedded into the multi-scale feature fusion process, strengthening the mapping between morphological characteristics and body weight. In the ECA-NL module, instance normalization, GLU gating, and attention threshold filtering were jointly employed to improve feature robustness under variable aquaculture imaging conditions and to enhance the expression of key morphological features. The synergy of the two modules improved prediction accuracy and robustness in complex aquaculture environments, providing a reusable technical approach for dual-view morphometric regression of aquatic organisms.

(3) The effectiveness of a three-stage collaborative pruning strategy for lightweight Transformers was validated on the current 30-individual dataset, and future work should extend this validation to larger, multi-source populations to confirm population-level generalizability. Through progressive compression combining attention head pruning, structured channel pruning, and depthwise separable self-attention replacement, a 16.7% reduction in parameter count was achieved with only negligible accuracy fluctuations. It was confirmed that collaborative pruning can simultaneously eliminate redundancy and provide structural regularization, offering solid empirical support for the efficient deployment of lightweight vision Transformers on aquatic edge-computing terminals.

Certain limitations remain in this study. The most critical limitation is the small number of independent biological replicates: all 1172 images were derived from only 30 fish, which constrains the biological diversity and poses a risk that individual-specific visual patterns may have contributed to the reported performance. Additionally, the dataset was constructed from a single farming environment and a single species; thus, the generalization capability across different geographic regions, culture modes, and closely related species requires further verification with larger, multi-source cohorts. The stability of feature extraction under extreme conditions such as high turbidity and rapid fish movement still has room for improvement. Future research can be extended in three directions: further reducing computational cost through model quantization and neural architecture search; constructing large-scale multi-environment, multi-species datasets to enhance generalization; and deeply integrating weight prediction with growth assessment and precision feeding to build a full-process intelligent perception and closed-loop decision system for intensive aquaculture.

In summary, this study demonstrates a promising lightweight prototype framework for stress-free visual estimation of live body weight in largemouth bass. Under the controlled tank conditions and limited sample size of this experiment, the model achieved competitive prediction accuracy with a compact parameter budget. However, this framework has not yet been validated in real production ponds or on actual edge devices. Future multi-site, large-cohort field trials and hardware-in-the-loop deployment tests are essential to confirm its practical viability. Methodological contributions are made in three dimensions—dual-view feature fusion, underwater anti-interference feature enhancement, and collaborative compression of lightweight Transformers—offering theoretical reference and technical support for the advancement of smart aquaculture toward precision, efficiency, and intelligence driven by deep learning.

## Figures and Tables

**Table 1:** Partitioning and statistics of the dataset.

Dataset	Number of Images	Body Length Range (cm)	Body Weight Range (g)
Training set	936	20.6–26.5	163–326
Validation set	236	20.5–24.7	150–260
Total	1172	20.5–26.5	150–326

**Table 2:** Image data augmentation and preprocessing pipeline and parameters (× indicates not applied; √ indicates applied).

Data Subset	Resize	Horizontal Flip (Probability)	Random Rotation	Color Jitter (Brightness, Contrast, Saturation, Hue)	Random Grayscale (Probability)	Normalization
Training set	224 × 224	0.5	±20°	(0.3, 0.3, 0.3, 0.1)	0.1	√
Validation set	224 × 224	×	×	×	×	√

**Table 3:** Basic hyperparameter settings.

Parameter Type	Setting Value
Optimizer	AdamW
Initial learning rate	0.0001
Batch size	16
Training epochs	200
Learning rate decay	Cosine annealing decay
Random seed	42

**Table 4:** Comparative experimental results of different models (Bold values denote the best performance under each evaluation metric).

Model	RMSE	MAE	MAPE	R^2^	Parameters (M)	FLOPs (G)
ViT	23.3262	15.1489	7.19%	0.5732	13.78	2.15
MobileNetV3-Large	27.4590	21.6677	9.61%	0.5380	17.23	1.20
EfficientNet	18.6052	15.0285	7.09%	0.7994	11.92	1.22
ResNet18	21.0143	13.9975	5.92%	0.7103	29.05	5.14
ResNet50	**15.1691**	**10.8684**	**4.73%**	0.8085	69.44	12.08
MobileViT-XXS-side	29.9048	24.2704	10.93%	0.4247	**3.38**	**0.46**
MobileViT-XXS-top	20.8111	14.5258	6.34%	0.7214	**3.38**	**0.46**
MobileViT-XXS	18.7820	12.3930	5.50%	0.7615	5.57	0.86
MEC-MobileViT (ours)	15.6546	11.0009	4.93%	**0.8424**	8.81	1.78
MECT-MobileViT (ours)	16.4201	11.1422	4.87%	0.8266	7.34	1.45

**Table 5:** Ablation experiment results for weight prediction (√ indicates the corresponding module is included in the model; × indicates the corresponding module is excluded).

MGMSF	ECA-NL	CAT	RMSE	MAE	MAPE	R^2^	Parameters (M)
×	×	×	18.7820	12.3930	5.50%	0.7615	5.57
√	×	×	16.3915	11.6997	5.29%	0.8272	8.81
×	√	×	18.5354	12.1493	5.15%	0.7790	5.57
√	√	×	15.6546	11.0009	4.93%	0.8424	8.81
√	√	√	16.4201	11.1422	4.87%	0.8266	7.34

**Table 6:** Five-fold fish-level cross-validation results of MECT-MobileViT.

Fold	RMSE (g)	MAE (g)	MAPE (%)	R^2^
1	17.5236	12.1034	5.32	0.8102
2	15.8923	10.8621	4.71	0.8341
3	16.9814	11.4872	5.08	0.8198
4	16.2407	11.0358	4.92	0.8255
5	17.8102	12.2451	5.41	0.8034
Mean ± SD	16.8896 ± 0.8176	11.5467 ± 0.6187	5.09 ± 0.29	0.8186 ± 0.0122

**Table 7:** Linear density distribution of the samples.

ID	Body Length (cm)	Body Weight (g)	x = Body Weight/Body Length (g/cm)
001	22.0	210	9.55
002	23.2	213	9.18
003	26.5	326	12.30
004	21.6	186	8.61
005	23.7	240	10.13
006	23.6	222	9.41
007	23.7	213	8.99
008	22.8	212	9.30
009	23.4	201	8.59
010	21.3	177	8.31
011	21.5	200	9.30
012	24.0	251	10.46
013	23.5	215	9.15
014	22.7	206	9.07
015	20.6	163	7.91
016	20.5	150	7.32
017	21.4	181	8.46
018	23.5	258	10.98
019	25.0	285	11.40
020	22.7	183	8.06
021	23.2	242	10.43
022	22.4	208	9.29
023	23.1	213	9.22
024	23.8	243	10.21
025	21.9	232	10.59
026	24.2	286	11.82
027	24.7	260	10.53
028	22.0	173	7.86
029	21.9	176	8.04
030	24.4	268	10.98

## Data Availability

Data are available on request, due to privacy.
